# Burden of Antibiotic Resistance in Hospitalized Children in Kenya: Associations with Mortality, Hospital Stay, and Treatment Costs

**DOI:** 10.4269/ajtmh.25-0440

**Published:** 2025-12-02

**Authors:** Veronicah Chuchu, Stanley Sayianka, Ita Teresa, Irene Inwani, Julius Oyugi, SM Thumbi, Sylvia Omulo

**Affiliations:** ^1^ Department of Medical Microbiology, University of Nairobi, Nairobi, Kenya;; ^2^ Washington State University Global Health Kenya, Nairobi, Kenya;; ^3^ Center for Epidemiological Modelling and Analysis, University of Nairobi, Nairobi, Kenya;; ^4^ FIND, Geneva, Switzerland;; ^5^ Commonwealth Pharmacists Association, London, United Kingdom;; ^6^ Kenyatta National Hospital, Nairobi, Kenya;; ^7^ Department of Paediatrics, University of Nairobi, Nairobi, Kenya;; ^8^ University of Nairobi Institute of Tropical and Infectious Diseases, Nairobi, Kenya;; ^9^ Institute of Immunology and Infection Research, University of Edinburgh, Edinburgh, United Kingdom;; ^10^ Paul G. Allen School for Global Health, Washington State University, Pullman, Washington

## Abstract

Antimicrobial resistance poses a growing threat to pediatric care, yet data on its clinical and economic burden in low-resource settings remain limited. The impact of antibiotic-resistant infections on mortality, hospital stay, and treatment costs was assessed among children admitted to a national referral hospital in Kenya in the present study. A retrospective review of medical records for pediatric patients (0–12 years) hospitalized with bacterial infections between January 2017 and December 2021 was conducted. Diagnoses included gastroenteritis, pneumonia, sepsis, urinary tract infections, meningitis, and others. Data on treatment, laboratory testing, outcomes, hospital stays, and costs were abstracted. Statistical analyses included Kaplan–Meier survival curves, Cox regression, and mixed-effects negative binomial and generalized linear models. Among 1,608 patients, 63% were infants, and 38% were referrals. Gastroenteritis (46%) and pneumonia (28%) were the most common diagnoses. Antibiotic-resistant infections occurred in 27% of participants and were associated with higher mortality (26% versus 9% in susceptible participants) and an attributable risk of 17%; the population attributable fraction was 65%. After adjustment, resistance was associated with increased mortality (HR 1.44), longer hospital stays (60% increase), and higher treatment costs (33% increase). Antimicrobial resistance significantly increases mortality, hospital stays, and healthcare costs in pediatric patients. Strengthening diagnostics, antimicrobial stewardship, and policy interventions is critical to address this threat.

## INTRODUCTION

Antimicrobial resistance (AMR) is a critical global public health threat, fueled by indiscriminate antimicrobial use, inadequate stewardship, and insufficient antibiotic development—factors that are modifiable and amenable to targeted interventions.^1^ Antimicrobial resistance compromises effective prevention and treatment of human and animal infections, impacting food safety and security, as well as health expenditure, morbidity, and mortality.^2^ Without action, AMR could cause 10 million deaths annually, reduce the global gross domestic product by 3.5%, and push 24 million people into poverty.^3^^–^^5^

Antimicrobial-resistant infections are associated with prolonged hospital stays, higher mortality, and increased healthcare and out-of-pocket expenditures.^6^^,^^7^ These indicators—mortality, hospitalization, and treatment cost—constitute the standard metrics for estimating AMR burden.^8^^,^^9^ The authors of a 2019 analysis attributed 1.27 million deaths to bacterial AMR, with the highest rates in western sub-Saharan Africa at 27.3 deaths per 100,000 population.^8^ The Global Leaders Group projects that AMR-related healthcare costs could reach $412 billion, with $443 billion in productivity losses.^10^

In recognition of the growing threat, the WHO launched a Global Action Plan in 2015.^11^ The 2024 United Nations High-Level Meeting reaffirmed commitments to reduce AMR-related deaths by 10% globally.^12^ Kenya aligned with the global framework and developed a National Action Plan, led by the Ministries of Health and Livestock, emphasizing multisectoral collaboration and evidence generation for AMR mitigation.^13^ Institutional responses include the establishment of the National Antimicrobial Stewardship Interagency Committee and the implementation of a One Health AMR Surveillance System that integrates data from human and animal health sectors.^14^

Despite these initiatives, Kenya faces data limitations that hinder the accurate assessment of the AMR burden.^15^^,^^16^ This impedes informed policy decisions and prioritization of interventions. Yet empirical estimates of AMR-related mortality, hospitalizations, and healthcare costs are critical for quantifying burden, evaluating diagnostics, and informing national investments.^17^^,^^18^ Improved diagnostics can reduce inappropriate antibiotic use, shorten hospital stays, and enhance patient outcomes by enabling the real-time identification of pathogens and resistance patterns.^17^

Robust burden estimates are essential to justify policy action, especially in contexts where the threat remains underrecognized.^18^ As AMR continues to impose rising costs through treatment, disability, premature death, and lost productivity,^3^ country-specific evidence is urgently needed to guide response strategies. To this end, the burden of antibiotic-resistant infections was assessed among hospitalized children in Kenya, with a focus on mortality, length of stay, and treatment costs. These metrics provide critical data to support the development of targeted, evidence-based interventions and national AMR policies.

## MATERIALS AND METHODS

### Study area, design, and population.

A retrospective longitudinal cohort study was conducted at Kenyatta National Hospital (KNH), a 1,800-bed national referral and teaching hospital in Nairobi, Kenya. As the country’s largest public healthcare facility, KNH provides tertiary pediatric care and receives referrals from across the country. It also has an in-house microbiology laboratory that performs routine antimicrobial susceptibility testing (AST).

The study population comprised pediatric patients aged 0–12 years admitted with bacterial infections between January 1, 2017 and December 31, 2021. This 5-year period was selected to achieve a sufficient sample size. Eligible diagnoses included urinary tract infections (UTIs), gastroenteritis, bacterial meningitis, sepsis, pneumonia, tonsillitis, pharyngitis, tracheitis, and wound infections.

### Sampling technique.

Patient files were identified using *International Classification of Diseases, 10th Revision* (ICD-10) codes corresponding to the above-specified infections. All files within the study period with ICD-10 codes of interest were reviewed for eligibility. Records were excluded if they fell outside the study period or study population, did not involve infections of interest, or were missing key data, including laboratory results (when AST was conducted), treatment sheets, invoices, patient outcomes, demographics, or admission histories. Patients admitted to the private wing were also excluded because data were missing in these files.

Infections were defined clinically or via laboratory confirmation. Cases were classified as resistant if 1) an AST indicated resistance to at least one prescribed antibiotic or 2) an AST was not performed but treatment was escalated to a higher antibiotic class after 48 hours, indicating therapeutic failure. This approach was guided by a point-prevalence survey conducted at the hospital, which revealed that only 34% of prescriptions were AST-supported.^19^ Patients hospitalized for <48 hours were excluded because resistance could not be reliably assessed.

Sample size determination was performed using Fisher’s exact test formula,^20^ assuming an AMR prevalence of 44% based on a previous study conducted at KNH.^21^ With 95% confidence and 5% precision, 379 records were targeted per year, totaling 1,895 records over 5 years. However, data were collected from all eligible files.

### Data collection and management.

Data from patient files were abstracted using a pretested REDCap tool (Vanderbilt University, Nashville, TN). Variables included demographics, diagnosis, outcomes, laboratory results, antibiotic regimens, treatment modifications, and direct medical costs, including pharmacy costs, antibiotic costs, culture and AST costs, and other laboratory test costs, as well as nursing, bed use, surgery, and other costs. Two days of training and a pilot ensured the tool’s reliability. Daily reviews of submitted records were performed to identify duplicates and resolve inconsistencies. Erroneous records were excluded.

## STATISTICAL ANALYSES

Data^22^ were analyzed using R (R Foundation, Vienna, Austria). Descriptive statistics were used to summarize patient characteristics. Continuous variables were presented as means or medians, whereas categorical variables were presented as frequencies and percentages. Mortality rates were compared between patients with resistant and susceptible infections. Attributable risk was calculated to quantify the incremental mortality associated with resistance, and population attributable fractions were computed to estimate mortality directly attributed to resistance.

Survival probabilities were evaluated using Kaplan–Meier survival estimates, stratified by resistance status and other relevant covariates. The survival function was estimated as follows^23^:

$$\hat{S}(t)=\prod_{i:t_{i}\leq t}\left(1-\frac{d_{i}}{n_{i}}\right)$$

where *t_i_* represents the time points at which deaths occurred, *d_i_* is the number of deaths at time *t_i_*, and *n_i_* is the number of patients at risk just before time *t_i_*. Group differences in survival were assessed using the log-rank test.

To explore factors associated with mortality, a Cox proportional hazard regression model was fitted. Predictor variables included resistance status, hospital referral, multiple infections, surgical procedures, bacterial sepsis, and age. The model included 1,608 patients, of whom 223 had observed deaths. The proportional hazards assumption was tested using the Schoenfeld residuals test. No significant violations were detected for surgery (χ^2^ = 0.013; *P* = 0.91), referral status (χ^2^ = 1.3258; *P* = 0.25), resistance (χ^2^ = 0.026; *P* = 0.87), or bacterial sepsis (χ^2^ = 1.348; *P* = 0.66). The global test also indicated no overall violation (χ^2^ = 2.4147; *P* = 0.66), confirming the model’s viability.

To examine treatment cost determinants, a generalized linear mixed model with a gamma distribution was used, whereas for the length of hospital stay, a mixed-effects negative binomial regression was used. Backward stepwise variable selection was applied, removing non-significant variables (*P* >0.20) while monitoring model performance using the Akaike information criterion (AIC) and the Bayesian information criterion (BIC). Variables were retained on the basis of statistical significance (*P* <0.05), model fit improvement, or theoretical relevance. Nested models were compared using likelihood ratio tests, with the final model selected on the basis of the lowest AIC/BIC, the highest log-likelihood, low deviance, and significant likelihood ratios.

Cost data were originally collected in Kenyan shillings (KSh) and converted to US dollars (USD), using an average exchange rate of 104.4 KSh per USD. This rate was derived from the mean annual exchange rates over the 5-year study period to ensure consistency and comparability.

## RESULTS

### Patient demographics.

Patient records were sampled from eligible admissions with bacterial infections between 2017 and 2021, yielding a final cohort of 1,608 pediatric patients. The majority (63%; *n* = 1,009) were infants under 1 year of age, and 57% (*n* = 923) were male. Referrals from other health facilities accounted for 38% (*n* = 608) of admissions, and 8% of the patients experienced multiple hospitalizations during the study period. The age distribution is illustrated in Supplemental Figure 1.

### Type of infections.

Among the 1,608 pediatric patients included in the analysis, 40% (*n* = 638) presented with multiple infections, resulting in a total of 2,287 recorded infection events. The most frequently diagnosed conditions were gastroenteritis (46%; *n* = 1,052), bacterial pneumonia (28%; *n* = 639), sepsis (12%; *n* = 265), and bacterial meningitis (11%; *n* = 239). Less common infections included UTIs (1.8%; *n* = 42), wound infections, pharyngitis, tracheitis, and tonsillitis.

Comorbid conditions were observed in 61% (*n* = 978) of all patients, with malnutrition being the most prevalent, affecting 24% (*n* = 232) of those with comorbidities. The temporal distribution of infections over the 5-year period is shown in Supplemental Figure 2.

### Antimicrobial resistance.

Among the 111 cases with AST results, resistance was defined on the basis of AST findings in 78% (87/111), whereas the remaining 22% (24/111) were classified on the basis of clinical therapeutic failure, as the antibiotics used for treatment were not included in the AST panel. Of the 87 AST-defined patients, 79% (69/87) had resistant infections, whereas 21% (18/87) had susceptible infections. Among the 1,521 cases assessed for clinical therapeutic failure, 24% (367/1,521) had resistant infections. Overall, 27% (436/1,608) of the total patient population had resistant infections. The temporal distribution over the 5-year period is shown in Supplemental Figure 3.

### Length of hospital stay.

Younger patients, particularly infants, remained hospitalized for longer than older children. Patients with antibiotic-resistant infections had a median length of hospital stay (LHS) of 12 days (mean = 14), compared with 7 days (mean = 9) for those with antibiotic-susceptible infections. Although the minimum stay was similar across groups (2 days), the maximum LHS was 125 days for patients with resistant infections and 172 days for those with susceptible infections.

The length of hospital stay also differed by clinical diagnosis. Patients with resistant meningitis and sepsis experienced the longest hospitalizations, with medians of 14 and 13 days, respectively. Those with gastroenteritis had median stays of 11 days, whereas children with UTIs had similar durations, despite a lower prevalence (Tables 1 and 2).

**Table 1:** Length of hospital stay summarized according to susceptibility

Infection	Meningitis	Pneumonia	Sepsis	Gastroenteritis	UTI
Resistant	28% (67/239)	37% (235/639)	31% (82/264)	24% (254/1051)	21% (9/42)
Susceptible median LHS	10 (mean = 11)	7 (mean = 7)	7 (mean = 7)	7 (mean = 7)	7 (mean = 7)
Resistant median LHS	14 (mean = 15)	12 (mean = 15)	13 (mean = 14)	11 (mean = 14)	11 (mean = 12)

LHS = length of hospital stay; UTI = urinary tract infection.

This table reveals that children diagnosed with meningitis, sepsis, and pneumonia experienced the longest stays.

**Table 2:** Length of hospital stay summarized according to pathogen

Pathogen	*E. faecalis*	*E. faecium*	*K. pneumoniae*	*S. epidermidis*
Resistant	43% (4/9)	36% (5/14)	17% (3/18)	24% (4/17)
Susceptible median LHS	8 (mean = 8)	9 (mean = 9)	9 (mean = 10)	6 (mean = 7)
Resistant median LHS	18 (mean = 23)	10 (mean = 14)	13 (mean = 12)	10 (mean = 10)

*E. faecalis* = *Enterococcus faecalis*; *E. faecium* = *Enterococcus faecium*; *K. pneumoniae* = *Klebsiella pneumoniae*; LHS = length of hospital stay; *S. epidermidis* = *Staphylococcus epidermidis*.

This table reveals that *E. faecalis* infections are associated with prolonged hospitalization.

### Predictors of LHS.

The baseline LHS for patients with susceptible infections and reference-level covariates was estimated at 8 days (95% CI: 7.12–8.15). Three factors strongly influenced LHS. Resistant infections increased the LHS by 60% (rate ratio [RR] = 1.60; 95% CI: 1.50–1.69), adding ∼5 days to the LHS. Meningitis extended the LHS by 23% (RR = 1.23; 95% CI: 1.14–1.32), and comorbidities extended the LHS by 10% (RR = 1.10; 95% CI: 1.04–1.17). A random intercept for patient ID (SD = 1.41; 95% CI: 1.36–1.47) accounted for inter-patient variability.

### Health outcome and death risk.

**Figure 1.**
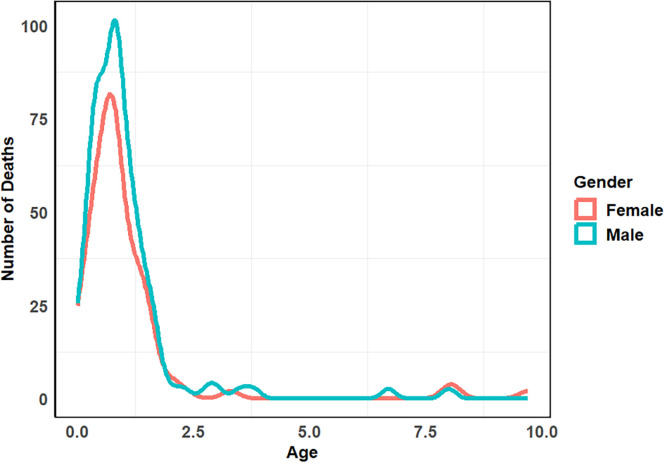
Age distribution of mortality among pediatric patients, stratified by sex. The plot reveals a high concentration of deaths in children under 1 year of age, with male infants experiencing a marginally higher mortality density compared with female infants.

**Figure 2.**
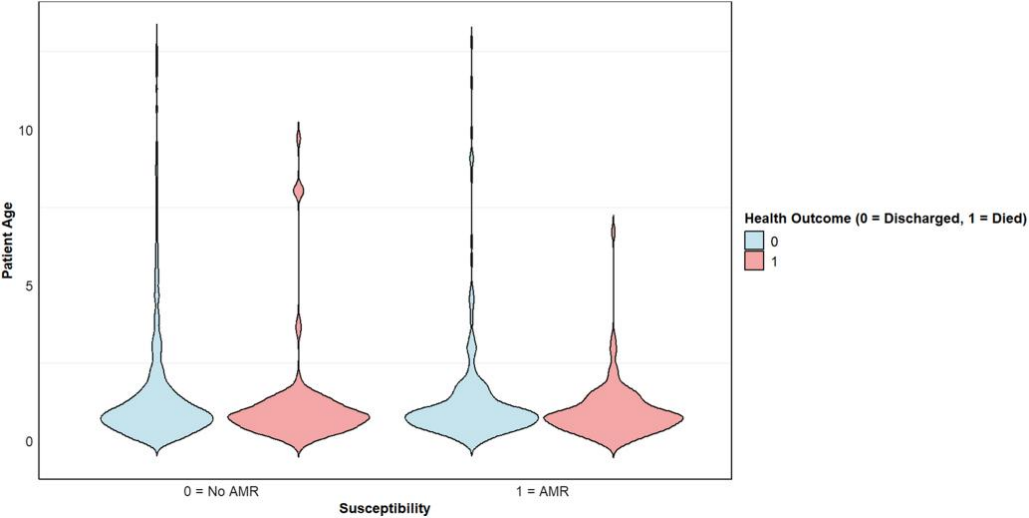
Violin plot revealing the interaction among age, antibiotic susceptibility, and patient outcomes. Most deaths occurred in younger patients across resistant and susceptible infection groups, with resistant infections associated with a more concentrated mortality distribution in infancy.

Among 1,608 hospitalized pediatric patients, 223 deaths (14%) were recorded, whereas 1,385 (86%) were discharged alive. Antibiotic-resistant infections were identified in 27% (*n* = 436) of patients, with a mortality rate of 26% (*n* = 113), compared with 9% (*n* = 110) among those with antibiotic-susceptible infections (*n* = 1,172). This corresponds to 260 versus 90 deaths per 1,000 patients, respectively. The attributable risk of AMR-related death was 17%, and the population attributable fraction was 65%, indicating that 73 of the 113 deaths in the resistant group were attributable to resistance. All AMR-related deaths occurred in children under 5 years of age, with infants (<1 year of age) disproportionately affected (Figures 1 and 2). The highest mortality rate was observed among patients diagnosed with sepsis (28%; *n* = 74/264), followed by meningitis (19%; *n* = 46/239) and pneumonia (18%; *n* = 113/639; Tables 3 and 4).

**Table 3:** Resistance and mortality status summarized according to infection

Infection	Gastroenteritis	Pneumonia	Sepsis	Meningitis	UTI
Resistant	24% (254/1051)	37% (235/639)	31% (82/264)	28% (67/239)	21% (9/42)
Mortality	122 (R = 58)	113 (R = 62)	74 (R = 44)	46 (R = 21)	1 (R = 0)

UTI = urinary tract infection; R = resistant.

This table reveals mortality by infection, with the greatest number of deaths occurring in sepsis cases.

**Table 4:** Resistance and mortality status summarized according to pathogen

Pathogen	*E. faecalis*	*E. faecium*	*E. coli*	*S. hemolyticus*	*K. pneumoniae*
Resistant	43% (4/9)	36% (5/14)	17% (2/12)	25% (2/8)	11% (2/18)
Mortality	2 (R = 2)	2 (R = 2)	2 (R = 1)	2 (R = 0)	1 (R = 0)

*E. coli* = *Escherichia coli*; *E. faecalis* = *Enterococcus faecalis*; *E. faecium* = *Enterococcus faecium*; *K. pneumoniae* = *Klebsiella pneumoniae*; R = resistant; *S. hemolyticus* = *Staphylococcus hemolyticus*.

This table reveals mortality by pathogen, with elevated antimicrobial resistance and mortality among patients with *E. faecium* and *E. faecalis* infections.

### Survival probability.

#### Kaplan–Meier estimate of survival function.

**Figure 3.**
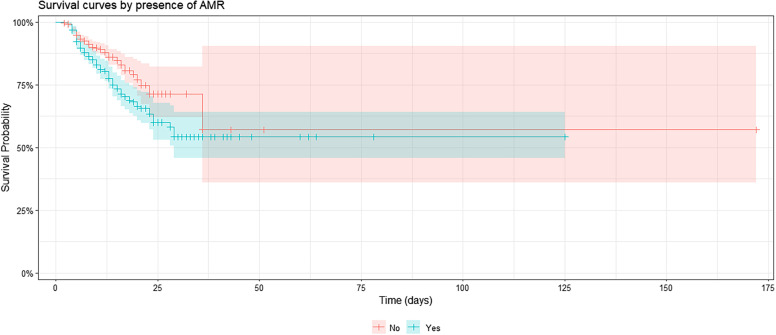
Kaplan–Meier survival curves comparing patients with antibiotic-resistant infections versus susceptible infections. Patients with resistant infections experienced reduced early survival, particularly within the first 25 days of hospitalization. Shaded areas represent 95% CIs.

The Kaplan–Meier survival analysis revealed a significantly lower survival probability among patients with resistant infections (log-rank χ^2^ = 12.3; *P* = 0.0005). By day 25, the survival probability declined to ∼60% for patients with resistant infections, compared with 70% for those with susceptible infections (Figure 3). Observed deaths in the resistant group (*n* = 113) exceeded expected deaths (88.7; [O − E]^2^/V = 12.3), whereas the susceptible group had fewer observed deaths (110) than expected (134.3).

Survival curves (Supplemental Figure 4) revealed that infants exhibited the lowest survival probability, especially early in hospitalization (log-rank χ^2^ = 19.1; *P* = 0.02). Infants with resistant infections had significantly worse outcomes, with 75 observed deaths versus 54.4 expected deaths (O − E)^2^/V = 10.97). Survival analyses revealed that vulnerable groups had worse outcomes. Referred patients had lower survival rates (log-rank χ^2^ = 48.5; *P* <0.0001), particularly those with resistant infections (66 observed deaths versus 38 expected deaths; Supplemental Figure 5). Surgical patients had steep early mortality rates (log-rank χ^2^ = 54.2; *P* = 0.0001), with 17 deaths in resistant cases versus five expected deaths ([O − E]^2^/V = 34.17; Supplemental Figure 6). Patients with sepsis had poor survival rates (log-rank χ^2^ = 60.6; *P* = 0.0001), especially those with resistant sepsis, who had 44 deaths versus 17 expected deaths ([O − E]^2^/V = 50.23; Supplemental Figure 7).

#### Cox proportional hazard regression.

The Cox proportional hazard regression (HR) confirmed that AMR was independently associated with increased mortality risk. Patients with resistant infections had a 44% higher hazard risk of death compared with those with susceptible infections (HR = 1.44; 95% CI: 1.09–1.90; *P* = 0.011). Other significant predictors included surgical procedures (HR = 2.27; 95% CI: 1.43–3.60; *P* = 0.0005), hospital referral (HR = 1.96; 95% CI: 1.49–2.58; *P* <0.001), and sepsis (HR = 1.93; 95% CI: 1.42–2.61; *P* <0.001). The model exhibited strong overall fit (likelihood ratio test: χ^2^ = 83.75, *P* <0.0001; Wald test: χ^2^ = 98.19, *P* <0.0001).

### Treatment cost.

Treatment costs were higher among children with antibiotic-resistant infections. The mean cost for resistant cases was $868 (median = $508), more than twice the cost of susceptible cases (mean = $376, median = $256). Resistant infections also had wider cost ranges ($105–$6,312 versus $42–$4,958). Daily treatment costs were significantly elevated, averaging $60.30/day for resistant infections versus $43.80/day for susceptible infections, with an added daily burden of $16.50. Cost variation over time is illustrated in Supplemental Figure 8.

By infection type, sepsis and meningitis were the most expensive to treat (mean = $762 and $679, median = $394 and $397, respectively), followed by pneumonia (mean = $620, median = $359) and gastroenteritis (mean = $444, median = $281). Resistant meningitis cases incurred nearly double the cost (mean = $1,059, median = $812) compared with susceptible cases (mean = $533, median = $325). Resistant sepsis was markedly more expensive to treat (mean = $1,457, median = $879) than susceptible sepsis (mean = $449, median = $297). Pneumonia also followed this trend (mean = $932 versus $439, median = $568 versus $285).

Among pathogens, resistant *Enterococcus faecalis* had the highest treatment costs (mean = $3,205, median = $3,466), far exceeding those of susceptible strains (mean = $241, median = $238). Resistant *Enterococcus faecium* also showed higher costs (mean = $820, median = $647) than susceptible strains (mean = $767, median = $370).

### Predictors of treatment cost.

**Figure 4.**
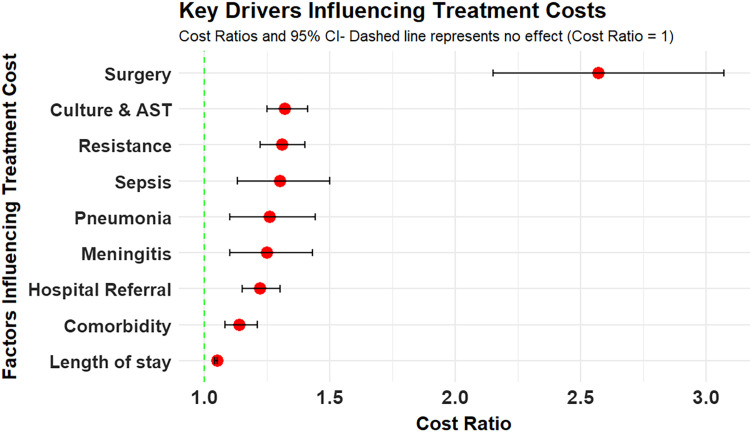
Key predictors of treatment cost among hospitalized children. Surgery was the strongest cost driver, followed by antibiotic resistance, diagnostic testing, sepsis, and other clinical factors. Error bars indicate 95% CIs; the dashed line marks the null effect (cost ratio = 1).

Antibiotic-resistant infections were associated with a 33% increase in total cost (cost ratio = 1.329; 95% CI: 1.243–1.421; *P* <0.001) compared with susceptible infections. Surgical procedures were the strongest cost driver, increasing costs by 152% (cost ratio = 2.521; 95% CI: 2.106–3.016; *P* <0.001). Other significant contributors included culture/AST (cost ratio = 1.326), hospital referrals (cost ratio = 1.221), and infections like sepsis (cost ratio = 1.308), pneumonia (cost ratio = 1.263), and meningitis (cost ratio = 1.253). Each additional hospital day increased cost by 5% (cost ratio = 1.047; 95% CI: 1.042–1.051; Figure 4).

## DISCUSSION

The substantial burden of antibiotic-resistant infections in hospitalized children, characterized by increased mortality, prolonged hospital stays, and elevated treatment costs, is demonstrated in the present study. The findings underscore the urgency of addressing AMR, particularly in resource-limited settings where diagnostic capacity is limited and empirical antibiotic use is common.

A key finding was the elevated mortality rate among patients with resistant infections—27% compared with 9% in those with susceptible infections—yielding an attributable mortality risk of 17% and a population attributable fraction of 65%. This aligns with the findings of previous studies from low- and middle-income countries (LMICs), which revealed that AMR doubles the risk of mortality (odds ratio = 2.8).^24^ Although the present study was limited to a single hospital, it offers important insights, especially because sub-Saharan Africa bears the highest AMR-related mortality burden.^8^ The Kaplan–Meier analysis revealed that mortality from antibiotic-resistant infections was highest early in hospitalization. In many LMICs, children who present with infections often receive empiric antibiotics to which pathogens may not be susceptible, leading to therapeutic delay and rapid clinical deterioration. This “gap period” between the initiation of empiric therapy and either the adjustment of antibiotics or receipt of susceptibility results likely contributes to the early mortality observed. Children under 5 years of age were the most affected, consistent with global data revealing that younger children are more vulnerable to severe infections,^25^ likely because of their immature immune systems and limited physiological reserves. Early mortality can also be linked to diagnostic underuse and limited access to timely diagnostics or second-line antimicrobials. The sharp early mortality gradient may also reflect system-level barriers. In the present study, 38% of children were referred from lower-level facilities, and referred patients with resistant infections had poor survival outcomes. Delays in recognition, transfer, and the initiation of appropriate treatment in referral pathways could be linked to early mortality. Taken together, these findings suggest that the early mortality burden of AMR in children may have been driven by both clinical (delayed effective therapy, infant vulnerability) and health system (limited diagnostics, referral delays) factors. Strengthening rapid diagnostic capacity, improving empiric treatment protocols, and streamlining referral systems are therefore critical to reducing early deaths attributable to resistant infections.

The current analysis also revealed sepsis as a major contributor to poor outcomes. Children with resistant bacterial sepsis had significantly lower survival probabilities. Globally, sepsis accounts for nearly 20% of all deaths, disproportionately affecting neonates, pregnant women, and patients in LMICs.^26^ The growing prevalence of drug-resistant Gram-negative bacteria in sepsis cases has exacerbated the problem.^27^ The limited availability of antibiotic therapies that can effectively combat these resistant pathogens, coupled with the rapid spread of resistance, has led to devastating outcomes for patients suffering from sepsis. Although there have been significant advances in the development of diagnostic technologies that could help diagnose sepsis and other bacterial infections, their availability and accessibility in low-resource settings remain challenging. In Kenya, only 64 of 1,037 laboratories are equipped for bacteriological testing,^28^ which hampers timely diagnosis and targeted therapy. The underutilization of diagnostic testing, particularly AST,^29^ likely influenced both clinical outcomes and the observed burden of AMR in the current study. Limited testing may have led to delays in identifying effective therapy, contributing to poorer outcomes, prolonged hospitalizations, and inflated treatment costs. Expanding diagnostic capacity, especially in district hospitals, is vital to improving patient outcomes.

Surgical patients with resistant infections exhibited significantly worse survival outcomes, with surgery independently associated with a 2.27-fold increase in mortality risk even after adjusting for confounders. Several factors may explain this observation. Surgical site infections and postoperative complications are more difficult to manage when they are caused by resistant organisms, often requiring prolonged hospitalization, additional surgical procedures, and extended or multiple antibiotic courses. These factors not only increase the risk of poor clinical outcomes but also substantially elevate treatment costs. Delays in initiating effective therapy, whether due to empiric regimens being inactive against resistant pathogens or awaiting AST results, further compound these risks, leading to deterioration in a group already vulnerable because of surgical stress and underlying illness. In addition, children who require surgery may represent more severe or complicated infections at baseline, amplifying both the clinical and economic burden of resistance. In referral cases, children referred from other hospitals often presented late, sometimes after receiving inadequate therapy, and may have carried multidrug-resistant pathogens acquired in those facilities. Such pathways contribute not only to higher mortality but also to greater costs associated with repeated investigations, surgery, prolonged treatment, and escalation to higher-class antibiotics.

Antibiotic resistance was also associated with prolonged hospitalization. Patients with resistant infections stayed a median of 5 days longer than those with susceptible infections. Similar trends were reported in Ghana and South Carolina, where resistant infections added 5–8 days to hospital stays.^30^^,^^31^ The negative binomial regression in the present study confirmed a 60% increase in length of stay for resistant infections. Extended hospitalization increases healthcare costs, strains limited resources, and imposes indirect socioeconomic burdens on families. Limited access to microbiology diagnostics and reliance on empirical therapy likely delay appropriate treatment, reinforcing therapeutic failure and prolonging care.

Resistant infections drove a 33% increase in treatment costs, reflecting additional medication needs. This aligns with previous studies that have revealed estimated increases in costs per patient due to AMR. Ghana, for example, has reported an additional $1,300 per AMR patient.^30^ In the present study, surgical procedures, hospital referrals, and infections such as meningitis, pneumonia, and sepsis significantly increased the total costs. Laboratory culture and susceptibility testing also contributed to a 33% increase, underscoring financial barriers to diagnostic stewardship. In resource-limited settings, unaffordable testing can lead clinicians to overuse broad-spectrum antibiotics, inadvertently driving resistance. Strengthening laboratory infrastructure, subsidizing diagnostics, and building clinician trust in laboratory data are essential for effective AMR control.

The current study has several limitations. First, being conducted at a single referral hospital may limit generalizability, as referral bias may skew the population toward more severe cases. Second, diagnostic testing was underutilized, which could lead to misclassification of therapeutic failures or underestimation or overestimation of resistant infections. Therapeutic change could also reflect clinician preference or non-resistance factors. Third, the retrospective design of the study limited the authors’ ability to access detailed laboratory protocols for specimen processing, contamination control, or criteria for distinguishing colonization from infection. This led to reliance on clinician diagnoses and AST reports documented in patient records, which may have introduced some degree of misclassification. Additionally, files lacking laboratory results in which cultures or AST had been requested were excluded from the study. This may have introduced sampling bias because records with missing results may reflect issues with access to diagnostics or record-keeping. Despite these limitations, the study findings highlight real-world challenges with AMR. Future research should be conducted to assess indirect AMR costs and evaluate diagnostics, stewardship, and prevention strategies across diverse settings to better understand pediatric AMR burden.

## CONCLUSION

The present study provides compelling evidence that antibiotic-resistant infections significantly contribute to higher mortality, longer hospital stays, and increased treatment costs among pediatric patients in Kenya. Although the findings are limited by the retrospective design and underutilization of diagnostic testing, they provide critical context-specific evidence on the burden of AMR. Enhancing diagnostic capacity, improving referral systems, and promoting rational antibiotic use are critical to mitigating the impact of AMR. The study findings emphasize the need for urgent, coordinated interventions that extend beyond policy frameworks to tangible health system improvements.

## Supplemental Materials

10.4269/ajtmh.25-0440 Supplemental Materials
