# Supplementary material for: Burden of Antibiotic Resistance in Hospitalized Children in Kenya: Associations with Mortality, Hospital Stay, and Treatment Costs
Source: Am J Trop Med Hyg. 2025 Dec 2;114(2):216–23. doi: 10.4269/ajtmh.25-0440 (PMC12874832; doi:10.4269/ajtmh.25-0440)
Supplement: Supplemental Materials [file tpmd250440.SD1.pdf]

Supplemental Materials

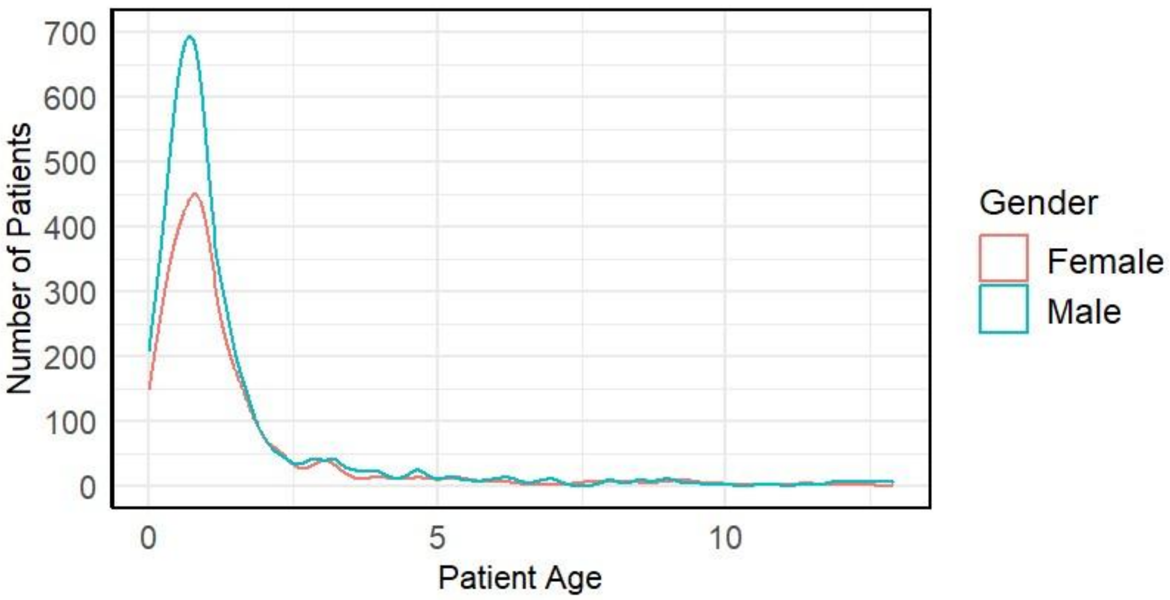

Figure S1: Age distribution of pediatric patients hospitalized with bacterial infections at KNH from 2017 to 2021, stratified by gender. Hospitalizations peak during infancy, with a gradual decline through older age groups. More male than female hospitalizations were recorded

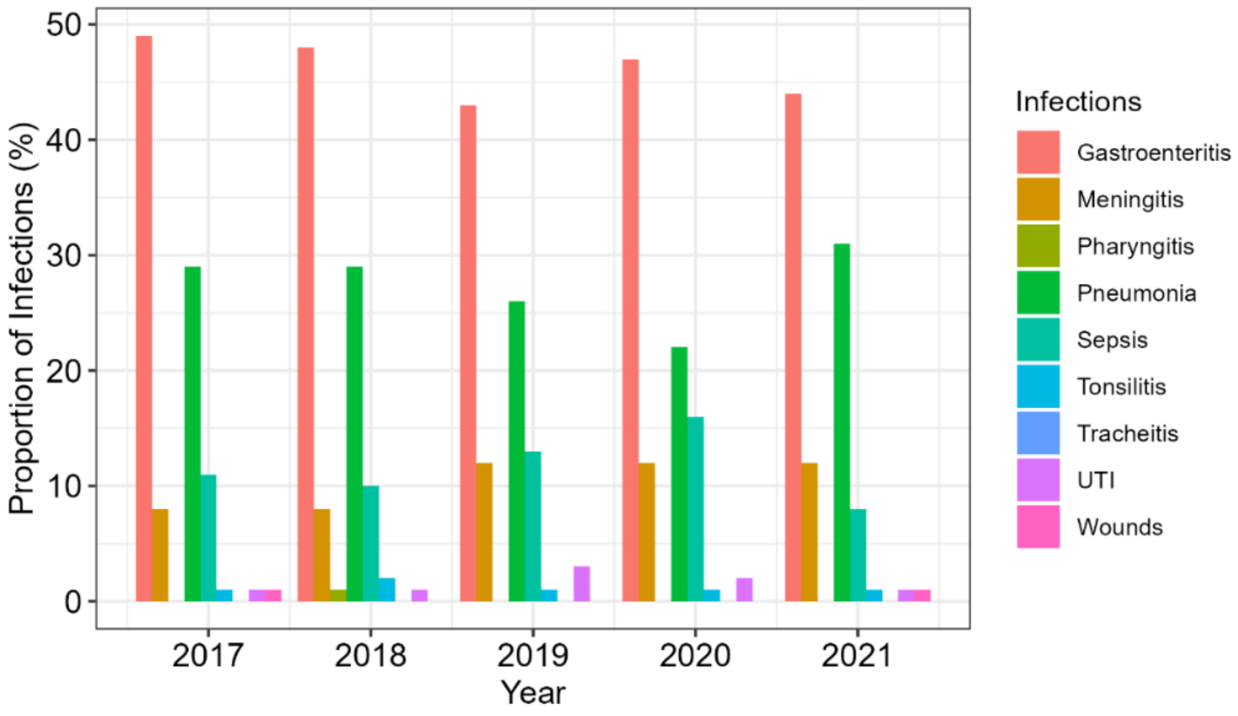

Figure S2: Proportion of bacterial infection types among hospitalized pediatric patients at KNH by year, 2017–2021. Gastroenteritis was the most common diagnosis across all years, followed by pneumonia and sepsis.

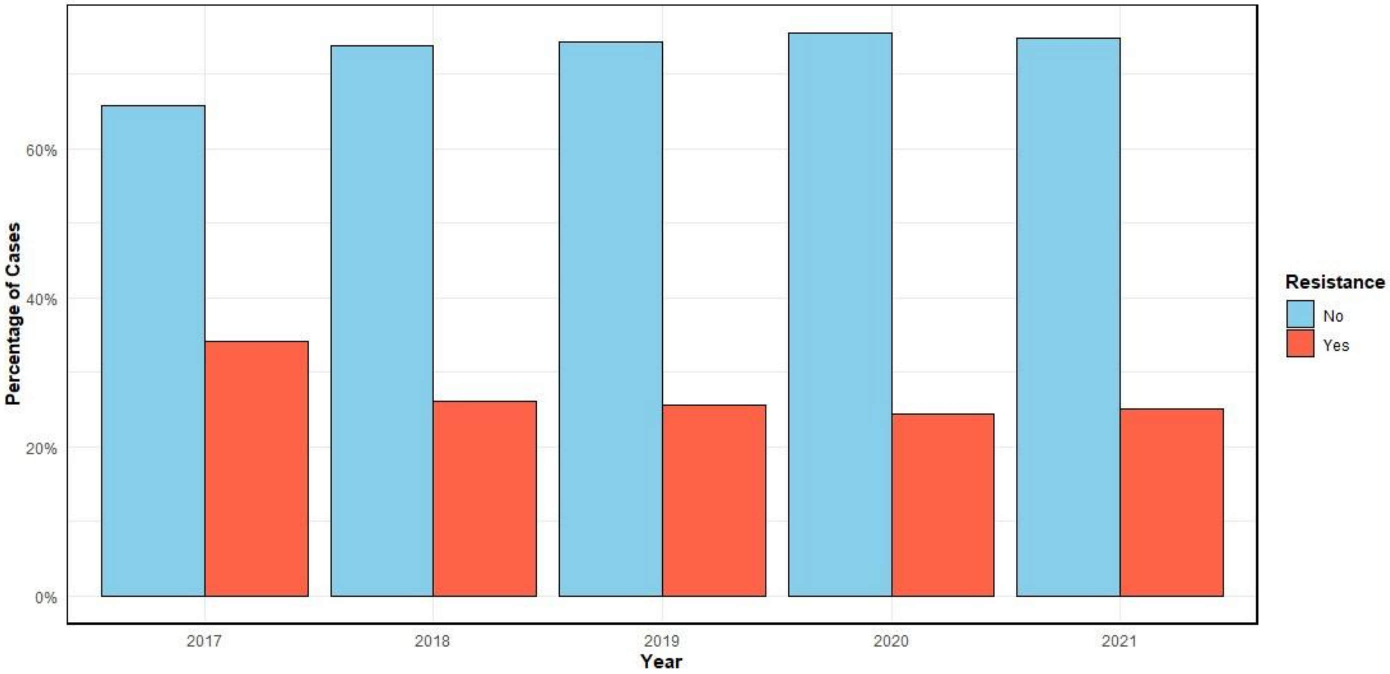

Figure S3: Proportion of pediatric patients with resistant infections at KNH by year, 2017–2021. 2017 and 2018 were the years with high cases of resistant infections at 34% and 26% respectively.

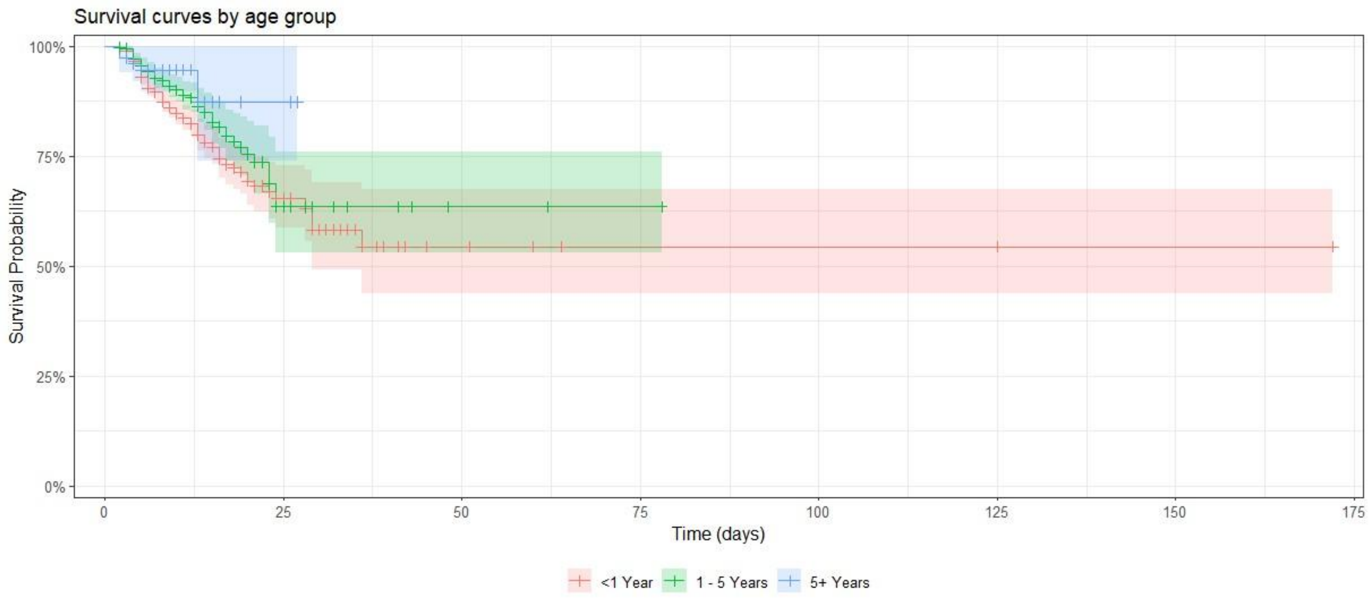

Figure S4: Kaplan–Meier survival curves by age group. Infants (<1 year) exhibited the lowest survival probability, particularly within the first months of hospitalization, while older children (5+ years) showed the highest overall survival.

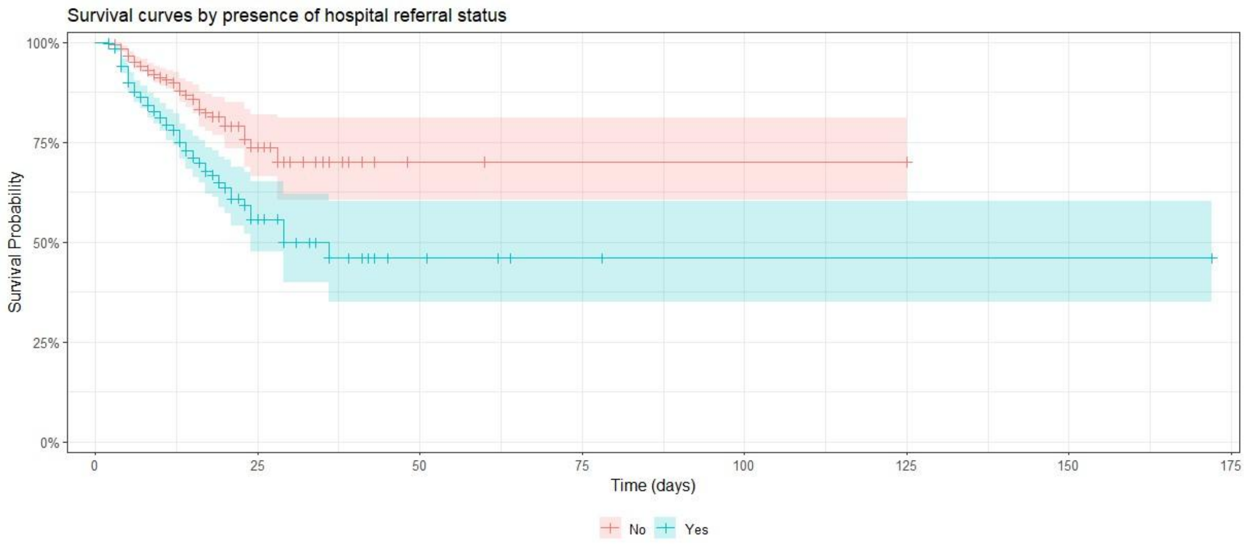

Figure S5: Kaplan–Meier survival curves by hospital referral status. Patients referred from other facilities exhibited significantly lower survival probabilities, particularly within the first month of admission, compared to non-referred patients.

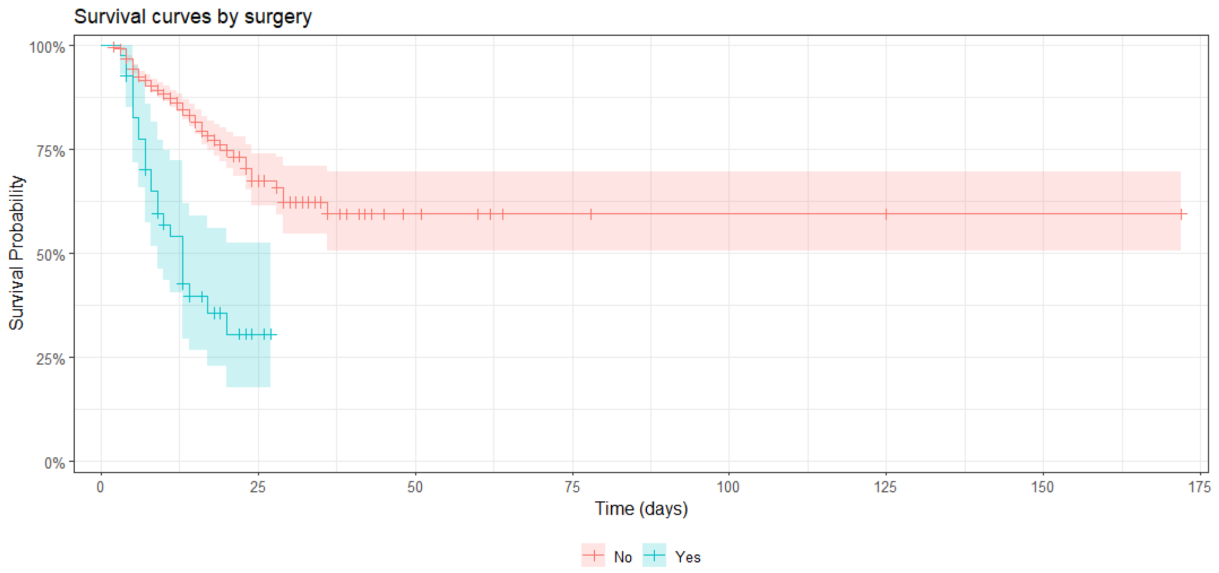

Figure S6: Kaplan–Meier survival curves by surgical status. Patients who underwent surgery had significantly lower survival probabilities, especially within the first 20 days of hospitalization, compared to non-surgical patients.

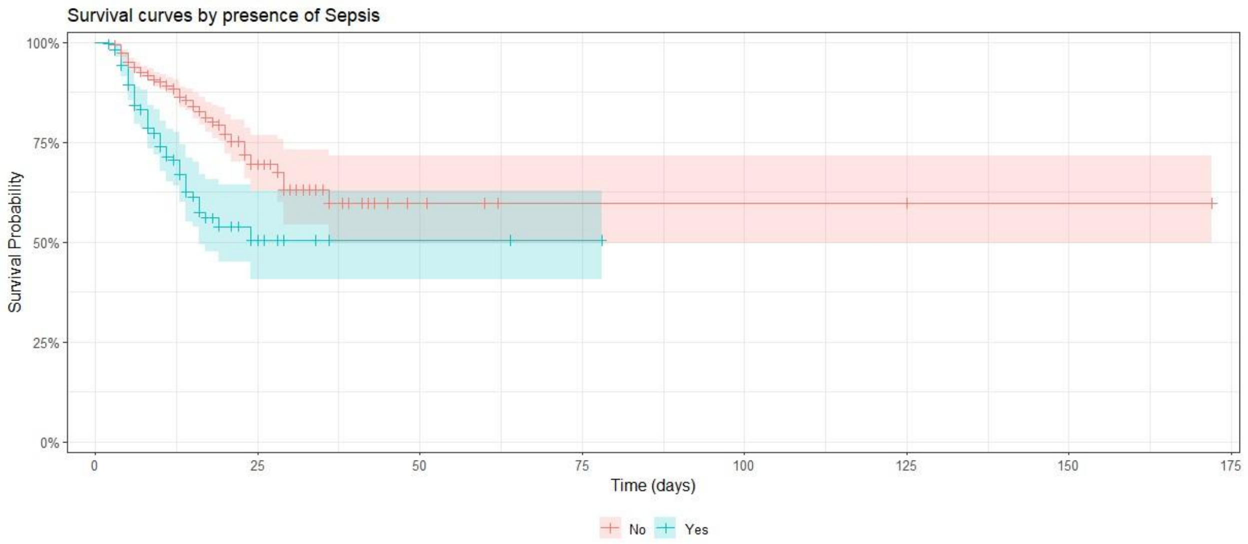

Figure S7: Kaplan–Meier survival curves by presence of sepsis. Patients diagnosed with sepsis experienced substantially lower survival probabilities, particularly within the first 30 days of hospitalization, compared to those without sepsis.

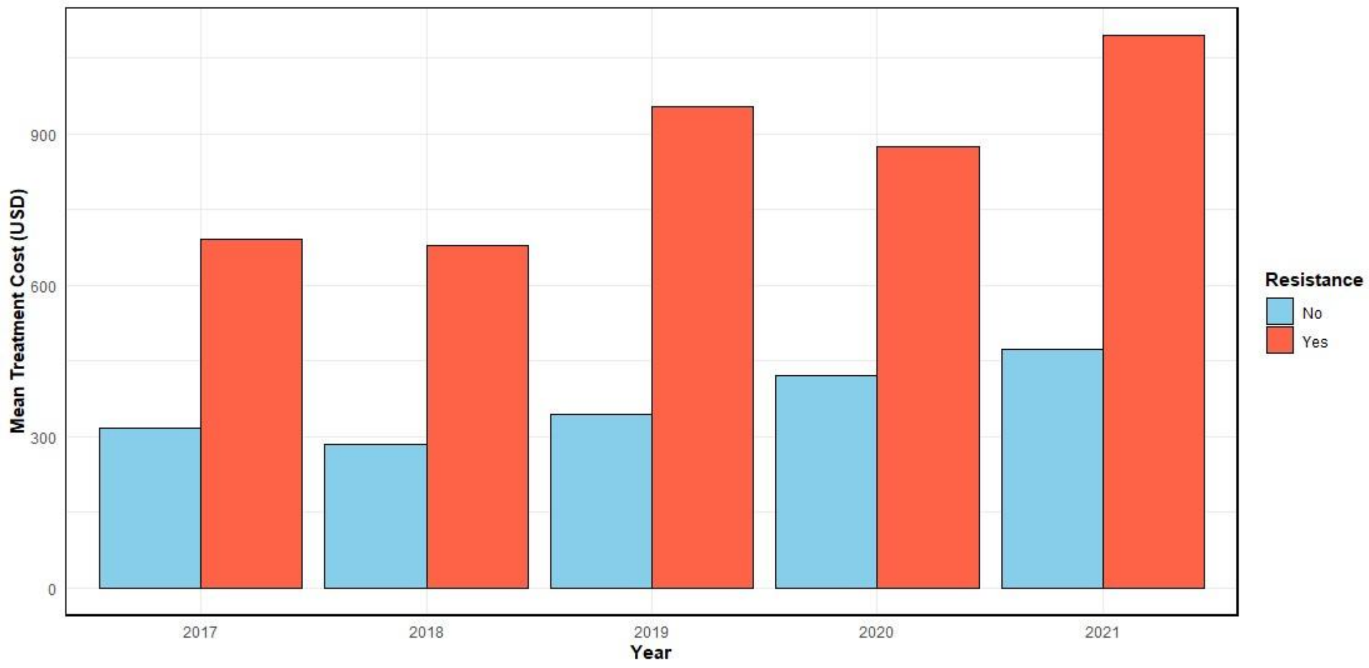

Figure S8: Variation in treatment cost of pediatric patients with resistant and susceptible infections at KNH by year, 2017–2021. 2021 and 20119 were the years with high treatment cost for resistant infections.
